# Commentary: Gluteal Region Reshaping of Massive Weight Loss Patients—A Decision-Making Strategy

**DOI:** 10.1055/s-0043-1777242

**Published:** 2024-03-04

**Authors:** Mohamed Ali Mahgoub

**Affiliations:** 1 Department of Plastic and Reconstructive Surgery, Faculty of Medicine, Mansoura University, Mansoura, Egypt

Gluteal Region Reshaping of Massive Weight Loss Patients—A Decision-Making Strategy

Aesthetic improvement of the buttocks involves either augmentation or contouring that may be obtained by liposculpture, surgical lifting, or combination. Patients with MWL have high expectations and are often treated with multiple procedures. Thus, an easy strategic approach personalized on each patient to treat multiple adjacent areas in one operation is necessary. Adipose tissue distribution, gluteal skin status, and BMI were the main factors that can forcefully affect our plan to guarantee reduction of unpleasant results and complications and improve patient satisfaction.



